# Supplementary material for: Isoflavonoid Profiling and Estrogen-Like Activity of Four Genista Species from the Greek Flora
Source: Molecules. 2020 Nov 24;25(23):5507. doi: 10.3390/molecules25235507 (PMC7727843; doi:10.3390/molecules25235507)
Supplement: Supplementary file 1 [file molecules-25-05507-s001.pdf]

molecules

MDPI

Supplementary material

Article

Isoflavonoid profiling and estrogen-like activity of four *Genista* species from the Greek flora

Antigoni Cheilari[1], Argyro Vontzalidou[1,2], Maria Makropoulou[1,2], Aggeliki K. Meligova[2], Nikolas Fokialakis[1], Sofia Mitakou[1], Michael N. Alexis[2*], Nektarios Aligiannis[1*]

[1] Department of Pharmacognosy and Natural Products Chemistry, Faculty of Pharmacy, National and Kapodistrian University of Athens, Panepistimiopolis, Athens, Greece; aligiannis@pharm.uoa.gr

[2] Institute of Chemical Biology, National Hellenic Research Foundation, Athens, Greece; mnalexis@eie.gr

* Correspondence: aligiannis@pharm.uoa.gr; Tel.: (+302107274757); mnalexis@eie.gr

Received: date; Accepted: date; Published: date

Abstract: As part of our ongoing research on phytoestrogens, we investigated the phytochemical profile and estrogen-like activities of eight extracts from the aerial parts of four *Genista* species of Greek flora using estrogen-responsive cell lines. The ethyl acetate and methanolic extracts of *G. acanthoclada*, *G. depressa, G. hassertiana* and *G. millii* were obtained with accelerated solvent extraction and their phytochemical profiles were compared using uHPLC-HRMS. Fourteen isoflavonoids, previously isolated from *G. halacsyi*, were used as reference standards for their identification in the extracts. Thirteen isoflavonoids were detected in both extracts of *G. acanthoclada* and *G. hassertiana*, while fewer and far fewer were detected in *G. millii* and *G. depressa,* respectively. The ethyl acetate extracts of *G. hassertiana* and *G. acanthoclada* displayed 2.45- and 1.79-fold higher, respectively, estrogen-like agonist activity in Ishikawa cells compared to MCF-7 cells at pharmacologically relevant concentrations. Both these extracts, but not that of *G. depressa*, contained mono- and di-*O*-*β*-D-glucosides of genistein as well as the aglycone, all three knowns to display full estrogen-like activity at lower-than-micromolar concentrations. The possibility of using preparations rich in *G. hassertiana* and/or *G. acanthoclada* extracts as potentially safer substitute for low-dose vaginal estrogen for menopausal symptoms is discussed.

Keywords: *Genista depressa*; *G. acanthoclada*; *G. millii; G. hassertiana*; isoflavones; estrogen-like activity.

Table S1: Structures of the identified isoflavonoids in *Genista* species extracts

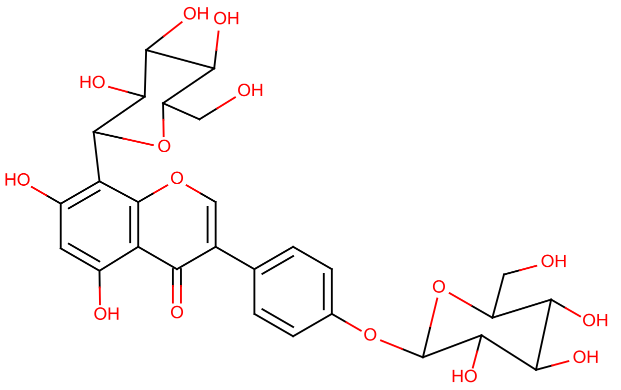	Compound **1:** 8-*C*,4'-*O*-diglucopyranosyl genistein
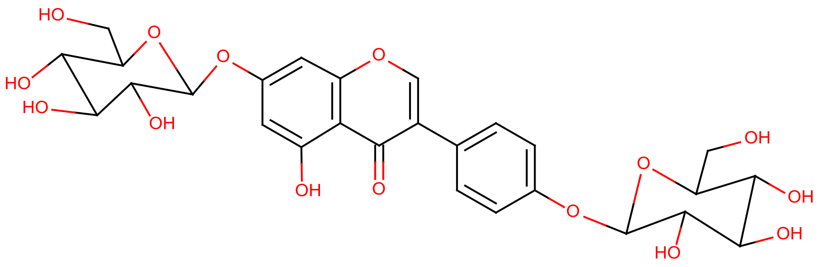	Compound **2:** 7,4'-di-*O*-glucopyranosyl genistein
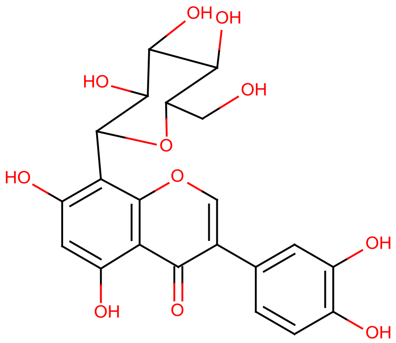	Compound **3:** 8-*C*-glucopyranosyl orobol
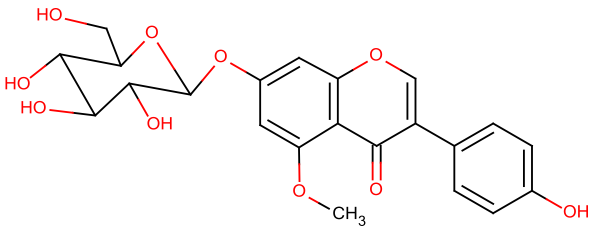	Compound **4:** 7-*O*-glucopyranosyl isoprunetin

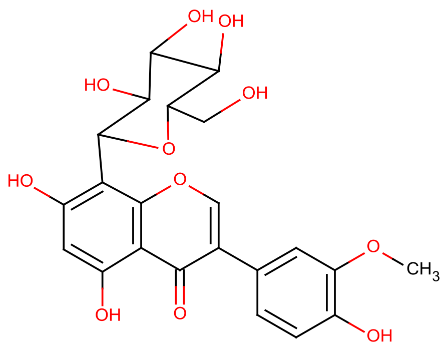	Compound **5:**<br><br>8-*C*-glucopyranosyl 3'-*O*-methylorobol
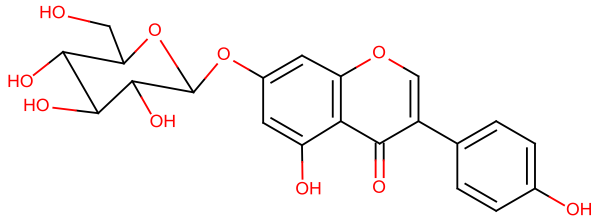	Compound **6:**<br><br>7-O-β-D-glucopyranosyl genistein
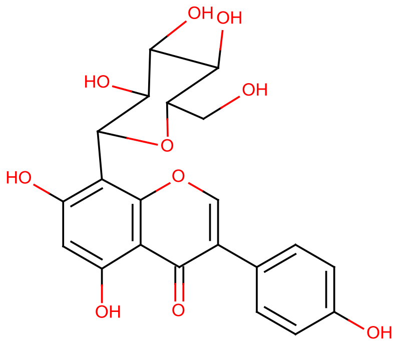	Compound **7:**<br><br>8-*C*-glucopyranosyl genistein
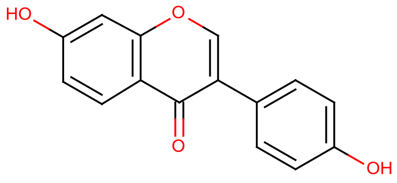	Compound **8:**<br><br>Daidzein
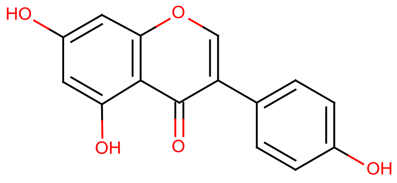	Compound **9:**<br><br>Genistein

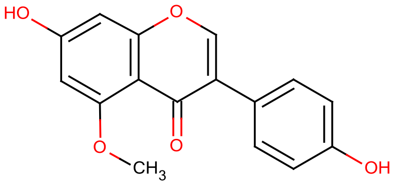	Compound **10:**<br><br>Isoprunetin
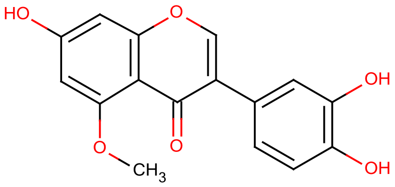	Compound **11:**<br><br>5-*O*-methylorobol
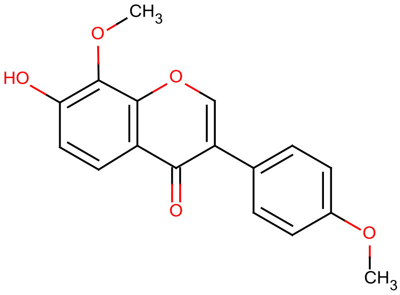	Compound **12:**<br><br>8- methoxy-formononetin
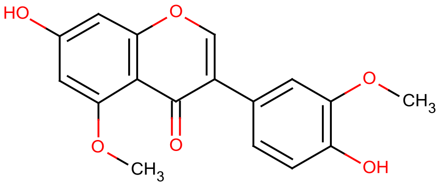	Compound **13:**<br><br>3'-methoxyisoprunetin
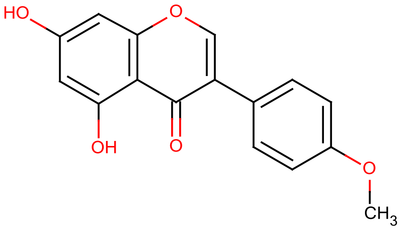	Compound **14:**<br><br>biochanin A

Table S2: Estrogen antagonist activity of *Genista* extracts

	**Alkaline phosphatase expression (Ishikawa cells) Antagonism[a] (% of ICI[b])**			**Cell proliferation (MCF-7 cells) Antagonism[a] (% of ICI[b])**
	**E2[c]+Extract (1 μg/mL)**	**E2[c]+Extract (0.1 μg/mL)**	**E2[c]+Extract (0.01 μg/mL)**	**E2[c]+Extract (1 μg/mL)**
**ICI182, 780**	100	100	100	100
***G. milii* - EtOAc**	M	M	M	M
***G.milii* - MeOH**	M	M	M	M
***G. acanthoclada* - EtOAc**	M	M	M	M
***G.acanthoclada* - MeOH**	M	M	M	M
***G. hassertiana* - EtOAc**	M	M	M	M
***G. hassertiana* - MeOH**	M	M	M	M
***G. depressa* - EtOAc**	M	M	M	M
***G. depressa* - MeOH**	M	M	M	M

[a] % antagonism = $(OD_{vehicle} - OD_{extract})*100/(OD_{vehicle} - OD_{ICI182,780})$. Values are Mean ± SEM of three independent experiments carried out in triplicate. Antagonist effects were classified as full, partial or weak depending on whether suppression of cell proliferation and alkaline phosphatase expression was ≥67%, 34–66% and 10–33% of that of ICI182,780. Effects <10% were classified as marginal (M). Estrogen-free cells were repleted with 0.1 nM estradiol

[b] ICI (ICI182, 780) was used at 0.1 μM
